# How do the physical and physiological demands of training and official football match for recreational players compare to those of semi-professionals?

**DOI:** 10.3389/fspor.2025.1553694

**Published:** 2025-05-16

**Authors:** Phornpot Chainok, Diogo Rego Abdul, Andreia Isabel Nogueira Pizarro, João Barreira, José Carlos Ribeiro, Maria Paula Santos, José Augusto Rodrigues dos Santos, João Ribeiro, João Ribeiro, Rodrigo Zacca

**Affiliations:** ^1^ Faculty of Sport Science, Burapha University, Saen Suk, Thailand; ^2^ Research Center in Physical Activity, Health and Leisure (CIAFEL), Faculty of Sports (FADEUP), University of Porto, Porto, Portugal; ^3^ Laboratory for Integrative and Translational Research in Population Health (ITR), Porto, Portugal; ^4^ Research Center in Sports Sciences, Health Sciences and Human Development (CIDESD), Vila Real, Portugal; ^5^ University of Maia, Maia, Portugal; ^6^ Centre of Research, Education, Innovation and Intervention in Sport (CIFI2D), Faculty of Sport (FADEUP), University of Porto, Porto, Portugal; ^7^ Research Center in Sports Sciences, Health Sciences and Human Development, CIDESD, CreativeLab Research Community, Vila Real, Portugal; ^8^ Department of Performance Optimization, GOD, Sporting Clube de Braga SAD, Braga, Portugal

**Keywords:** exercise, health, heart rate, football, cardiorespiratory fitness, training load

## Abstract

Playing football at very different competitive levels can lead to different physical and physiological demands. Daily routines of these practitioners can also show differences in terms of leisure time, main professional occupations, among other activities. Therefore, understanding the physical and physiological demands between recreational and Semi-Professional players is crucial for designing appropriate training programs and assessing potential health benefits. We compared physical and physiological demands, particularly, considering external load at different heart rate (HR) intensity zones, between recreational and Semi-Professional football players by means of an ecological approach. We evaluated internal and external load related variables during i) a typical week of training and ii) a 90-min 11-vs.-11 official football match in recreational (*N* = 9) and Semi-Professional teams (*N* = 7). The measures were collected using wearable technologies (high-frequency GPS tracking and inertial devices). Semi-Professional players performed more training sessions·week^−1^ (4 vs. 2) and are likely to be involved in 26.2% higher weekly vigorous physical activity volume (min·week-1) (95%CI: 49.2–87.5 min·week-1; *p* = 0.050; ɳp^2^ = 0.175; small effect) than recreational practitioners, despite any other type of professional occupation. Mean pre-match baseline HR was 17% lower in Semi-Professional than recreational group (*p* = 0.003; ɳp^2^: 0.475; moderate effect). Likewise, mean HR reserve was 12% higher in semi-professional than recreational players (*p* = 0.002; ɳp^2^: 0.–0.551; moderate effect). Mean HR values during 90 min 11-vs.-11 official football match were 80 ± 6%HRmax (Semi-Professionals; *N* = 7) and 81 ± 5%HRmax (recreational; *N* = 9), respectively (diff: −1%; 95%IC: −7.8 to 4.9%; *p* = 0.630; ɳp^2^: 0.017). Semi-Professional players covered 41% more distance at high HR (>85% HRmax) (95% CI: 211–5,103 m, *p* = 0.035, *η*² = 0.279; moderate effect) during 11-vs.-11 official football match, suggesting greater cardiorespiratoy fitness when compared to recreational players. At last, the distance covered at 70%–80% HR level was positively associated with the % at very vigorous physical activity levels in training (*p* = 0.033; *r* = 0.533). These findings suggest that recreational players may require modified training protocols to optimize performance while managing internal load.

## Introduction

Football practice is suggested as a good option of physical activity or exercise for health promotion (1–3), and characterized by intermittent efforts at different intensities and durations (4–9), requiring technical, tactical, physical, mental and social skills, among others (10–13). Some authors suggest that only two football sessions (60 min each) per week (over 12–16 weeks) produces relevant physiological adaptations in the human body (14). In fact, football is a collective sport with many physiological benefits in relation to some health-related markers (∼10% decrease in resting heart rate and ∼13% of total body fat) (14). The stimulus for this football training induced adaptations is the physiological stress (i.e., internal load) imposed in the athletes by the external load (8, 9, 15). In this sense, understanding the dose-response relationship between internal and external load is crucial to the adaptational changes and/or health implications. However, studies exploring and comparing external and internal load related variables in football players of different levels are scarce (11, 16, 17).

Authors suggest that individuals with a higher level of physical fitness in football, present lower values of internal load (e.g., heart rate) for the same external load, that is, for the same task, there is a lower effort on the part of those who are more able and, consequently, train more (duration and frequency) (3, 16–19). The adaptations generated by external and internal loads are expected to be different between levels. Given the rising participation in recreational football, understanding how its physical demands differ from semi-professional levels is crucial for injury prevention and performance optimization. Besides, given the importance of verify the impact of football practice on the cardiorespiratory fitness of its practitioners (9), the aim of this study was to compare physical and physiological demands, particularly, considering external load at different hear rate intensity zones, between recreational and Semi-Professional football players by means of an ecological approach. We hypothesize that semi-professional football players will exhibit a higher volume of weekly vigorous physical activity during training compared with recreational players and will also cover greater distances at high heart rates, suggesting superior cardiorespiratory fitness.

## Materials and methods

### Participants

This is a convenience sample, selected based on accessibility and availability and was composed by 30 (*N* = 30) football players who were evaluated in internal and external load related variables during (i) a typical week of training and (ii) a 90 min 11-vs.-11 official football match in two distinct levels: Semi-Professional (*N* = 15) and recreational (*N* = 15). The football players from the Semi-Professional level were recruited from a team competing at League 3 (3rd national League of the Portuguese Football Federation), while the recreational players were recruited from the Viseu county championship—Constructions Pelezinhos, LDA of the Viseu Football Association (Portugal). However, 14 players were excluded from the study (∼47% of the total sample) for spending less than 10 h in 4 days with their accelerometers during typical week of training and recovery routine (20) and two due to non-attendance at the time of assessment and placement of accelerometers and non-response to contact. Thus, nine (*N* = 9) Semi-Professional football players (age: 23.6 ± 2.2 year; height: 1.76 ± 0.9 m; weight: 69 ± 8.3 kg) and seven (*N* = 7) recreational players aged between 18 and 35 years (26.1 ± 6.7 year; height 1.77 ± 0.72 m; weight 73 ± 4.8 kg) were fully evaluated in the current study (*N* = 16). All participants were healthy, without any serious musculoskeletal, metabolic, cardiorespiratory, hematological, or endocrine exercise disorders in the previous 6 months.

#### Procedures

Players were informed about the objectives, risks and discomforts of the study and gave their informed consent for participation. The Semi-Professional (SP) team (Portugal League 3) participated in 4 training sessions and 1 match during the study week. All the sessions occurred in the morning (9:30 AM). The recreational (RC) team (Viseu county championship) participated in 2 training sessions and 1 match during the study week. All the sessions occurred in the evening (8:00 PM), after a workday. The training sessions in the SP group averaged 103.5 ± 10.6 min and 49.2 ± 14.7 min in the RC group. The study was conducted in accordance with the Declaration of Helsinki and approved by local university). Informed consent was obtained from all subjects involved in the study.

**Figure 1:**
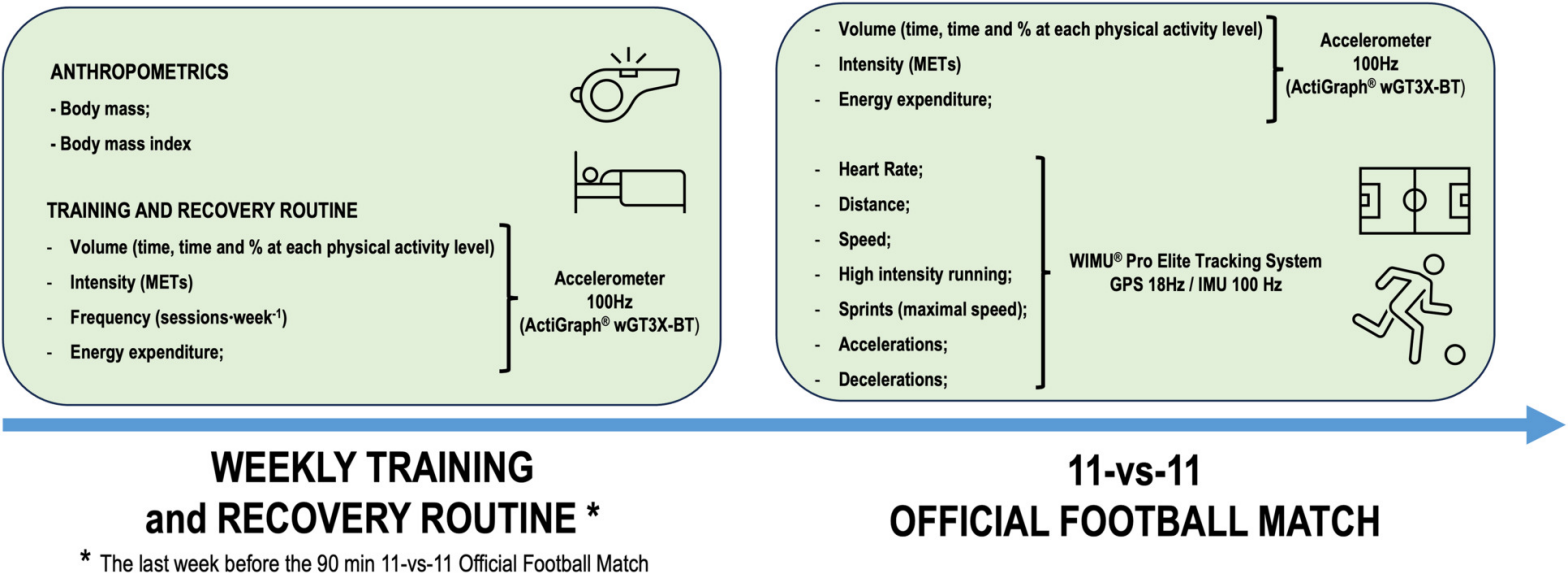
Study design.

We compared the physical and physiological demands between recreational and Semi-Professional football players during (i) a weekly training and recovery routine; and (ii) during a 90 min 11-vs.-11 official football match (see Figure 1):

i. Weekly training and recovery routine: Standardized methodologies were used to quantify the training load of a typical week of the competitive period in both teams (21), i.e., the last week before the 90 min 11-vs.-11 official football match.
ii. 90 min 11-vs.-11 official football match: the evaluations during the match of both teams occurred at the same time of the day (15h00). In relation to the recreational team, the match took place in a dirt field of 100 × 58 m with air temperature of ∼23°C and relative humidity of ∼65%. In relation to the Semi-Professional team, the match took place on a natural grass pitch of 105 × 68 m with air temperature of ∼27.5°C and relative humidity of ∼50%.

Physical and physiological variables were recorded using scales (Tanita InnerScan V BC-545N, Tokyo, Japan); stadiometer (Seca 213, Hamburg, Germany); accelerometers (Actigraph wGT3X-BT, California, USA) and GPS (WIMU Pro_1219, Almería, Spain), the statistical data accelerometry data was analysed with Actilife v6.3 (California, USA), and Troiano cutpoints were used to measure physical activity levels respectively.

The values of body mass (kg), height (cm) and body mass index (BMI) of the practitioners were recorded up to 48 h before the 90 min 11-vs.-11 official football match by controlling feeding and hydration.

Weekly training routine: Both teams were monitored with one accelerometer (Actigraph wGT3X-BT, Florida, USA, 100 Hz) for each player to quantify sedentary habits and level of physical activity during training and recovery periods. During 90 min 11-vs.-11 official football match: Each athlete used an accelerometer (Actigraph wGT3X-BT, Florida, USA, 100 Hz) to estimate energy expenditure (kcal); MET (rates); intensity of physical activity (Sedentary-ST, light-LPA, moderate-MPA, vigorous-VPA or moderate-vigorous-MVPA) and respective percentage (20). Match-play physical demands were recorded using global positioning systems (GPS) technology with a sampling frequency of 18 Hz and inertial movement unit (IMU) accelerometer with a sampling rate of 100 Hz (WIMU Pro_1219, Almeria, Spain, https://www.hudl.com/en_gb/products/wimu). The devices were fitted to the upper back (i.e., between the scapulae) of each player in an adjustable neoprene harness as recommended by the manufacturer. All devices were switched on in an outdoor environment and ∼10 min were given to acquire satellite connection. Each player used a HR monitor chest belts (GARMIN^TM^, Garmin Ltd., Olathe, KS, USA) attached to the ribcage under the musculus pectoralis major and coupled to the wearable tracker (WIMU Pro_1219, Almeria, Spain, https://www.hudl.com/en_gb/products/wimu) to monitor heart rate (maximum, average, absolute, relative, and specific zones) during the 1st and 2nd half of the match. Baseline HR (heart rate up to 20 min before the match) and HR reserve were also measured, based on baseline HR as suggested by Karvonen and Vuorimaa (22).

#### Statistical analysis

The Shapiro–Wilk test was employed to assess data normality. Multifactorial ANOVA was conducted, with the “team” variable serving as the factor division, and Bonferroni *post hoc* tests were used for inter-team comparisons. Significant differences between groups were assumed at a 5% alpha level. Regarding effect size, eta-squared (*η*^2^) was used to quantify the percentage of the variance explained (effect size) and interpreted as follows: 0 < *η*^2^ < 0.04 “trivial”, 0.04 ≤ *η*^2^ ≤ 0.24 “small”, 0.25 ≤ *η*^2^ < 0.64 “moderate”, and *η*^2^ ≥ 0.64 “large.” (23)*.* Potential associations between variables were examined using Pearson correlation tests (assuming correlation exists when *p* ≤ 0.05) for normally distributed variables and Spearman correlation tests (assuming correlation exists when *p* ≤ 0.01) for non-normally distributed variables. Interpretation criteria were as follows: 0.00 < *r* or *ρ* < 0.10 indicated “negligible correlation”; 0.10 < *r* or *ρ* < 0.39 indicated “poor correlation”; 0.40 < *r* or *ρ* < 0.69 indicated “moderate correlation”; 0.70 < *r* or *ρ* < 0.89 indicated “strong correlation”; and 0.90 < *r* or *ρ* < 1.0 indicated “very strong correlation”. The IBM Statistical Package for the Social Sciences (SPSS) 29.0 was utilized for all analyses, and a significance level of 0.05 was assumed.

## Results

Table 1 (weekly training and recovery routine) shows the mean values and standard deviations for age, weight, energy expenditure and weekly time of physical activity, in their absolute and relative form for both Semi-professional and recreational teams (*N* = 22). All values were similar between groups, except for body mass index (BMI) (diff: −1.7 kg·m^−2^; 95%IC: −2.7 to −0.5 kg·m^−2^; *p* = 0.006; ɳp^2^: 0.316; moderate effect**)** and total weekly time in vigorous PA (diff: 24.3 min· week^−1^; 95%IC: 49.2–87.5 min· week^−1^; *p* = 0.050; ɳp^2^: 0.171; small effect**)**. At this level, the Semi-Professional team performed, on average, approximately +24 min of vigorous physical activity when compared to the recreational team. The accelerometer data analyzed the training sessions of each team and all other parts of the day of the practitioners.

**Table 1:** Weekly training and recovery routine: anthropometrics, metabolic and physical activity of the 22 subjects analyzed (*N* = 22).

	Team level	Mean	SD	Mean Diff. (Δ%)	95% IC		ɳp^2^	*p*-value
					Lower	Upper		
Weight (kg)	SP	69	8.3	−3.8 (−5.5)	68.2	78.0	0.071	0.230
	RC	73	4.8					
Age (years)	SP	24	2.2	−2.5 (−10.6)	22.9	29.3	0.073	0.224
	RC	26	6.7					
BMI (kg·m^−2^)	SP	22	1.5	−1.7 (7.2)	−2.7	−0.5	0.316	**0.006** *
	RC	24	0.7					
Total Energy Expenditure (kcal)	SP	5,151	1,171.6	244.4 (4.7)	4,020.9	5,792.2	0.010	0.663
	RC	4,907	1,413.1					
Energy expenditure (kcal·day^−1^)	SP	736	167.4	122.6 (16.7)	494.3	732.4	0.120	0.114
	RC	613	176.7					
Energy Expenditure (kcal·h^−1^)	SP	53	10.8	2.4 (4.5)	43.1	58.4	0.012	0.624
	RC	51	11.3					
PA mean intensity (MET's)	SP	1	0.1	0.0 (0.0)	1.3	1.471	0.036	0.398
	RC	1	0.1					
Total sedentary time (min·week^−1^)	SP	4,278	611.9	130.6 (3.1)	3,756.2	4,538.4	0.014	0.598
	RC	4,148	478.8					
Light PA (total) (min·week^−1^)	SP	485	111.3	−25.8 (−5.3)	413.5	607.6	0.009	0.675
	RC	511	173.6					
Moderate PA (total) (min· week^−1^)	SP	371	82.8	5.0 (1.3)	295.1	436.7	0.001	0.910
	RC	366	125.1					
Vigorous PA (total) (min week^−1^)	SP	93	26.5	24.3 (26.2)	49.2	87.5	0.171	**0.050** *
	RC	68	29.2					
Very vigorous PA (total) (min·week^−1^)	SP	30	10.3	9.1 (30.8)	12.7	28.1	0.152	0.073
	RC	20	12.2					

SP, semi-professional level; RC, recreational level; BMI, body mass index; PA, physical activity; min·week^−1^, minutes per week; kcal·day^−1^, kilocalories per day; kcal·h^−1^, kilocalories per hour; MET'S-metabolic equivalent.

* Significant difference; effect sizes (eta squared, *η*^2^): 0 < *η*^2^ < 0.04 “trivial”, 0.04 ≤ *η*^2^ ≤ 0.24 “small”, 0.25 ≤ *η*^2^ < 0.64 “moderate”, and *η* ^2 ^≥ 0.64 “large.”

Table 2 shows the relative values of intensity of physical activity during the 90 min training sessions of the 16 subjects fully evaluated in the current study (*N* = 16). Most of the percentage values were similar between groups, except for the percentage of very vigorous physical activity (*p* = 0.049; ɳp^2^: 0.175; small effect). The Semi-Professional group has on average, ∼0.2% higher percentage of physical activity time at the very vigorous level, compared to the recreational level.

**Table 2:** Characteristics (intensity and percentage) of the practice of PA (in training sessions) of the 16 subjects fully evaluated in the current study (*N* = 16).

	Team level	Mean	SD	Mean Diff. (Δ%)	95% IC		ɳp^2^	*p*-value
					Lower	Upper		
% Light PA (training)	SP	9	1.3	−1 (−6.5%)	8.5	11.1	0.031	0.430
	RC	10	2.5					
% Moderate PA (training)	SP	7	1.4	0 (0.0%)	5.9	8.2	0.000	0.964
	RC	7	1.9					
% Vigorous PA (training)	SP	2	0.6	1 (27.8%)	0.9	1.7	0.166	0.060
	RC	1	0.5					
% Very vigorous PA (training)	SP	0.6	0.2	0.2 (33.3%)	0.3	0.5	0.175	**0.049** *
	RC	0.4	0.2					
% Moderate-vigorous PA (training)	SP	10	1.7	1 (7.4%)	7.4	10.2	0.030	0.442
	RC	9	2.3					
Moderate-vigorous PA (total) (min·week^−1^)	SP	493	97.7	38 (7.7%)	368.8	540.7	0.025	0.486
	RC	455	154.5					

SP, semi-professional level; RC, recreational level; PA, physical activity; min·week^−1^, minutes per week.

* Significant difference; the remaining percentage refers to time spent in sedentary behavior. Effect sizes (eta squared, *η*^2^): 0 < *η*^2^ < 0.04 “trivial”, 0.04 ≤ *η*^2^ ≤ 0.24 “small”, 0.25 ≤ *η*^2^ < 0.64 “moderate”, and *η*^2^ ≥ 0.64 “large.”

From the total volume of weekly physical activity of the SP group, 6.8 ± 1.0% of the time was occupied by training and match. In the RC group, only 5.1 ± 0.7% of the total weekly PA was occupied by football practice moments. In absolute time, this period is equivalent to approximately 7 h of practice in the SP group and 4 h in the RC group. The difference found was already expected, since the SP group performed 4 practices and 1 match and the RC group performed only 2 practices and 1 match.

Table 3 shows physical performance for both groups during a 90-min 11-vs.-11 official football match, where differences were observed between SP and RC for acceleration and deceleration load (AU), distance (m) covered during high accelerations (>3 m.s^−2^) and decelerations (≤3 m.s^−2^), and the number of high accelerations and decelerations (*p* = 0.000–0.050; ɳp^2^: 0.244–0.520; small to moderate effect).

**Table 3:** Comparison between SP and RC groups of physical performance values during 90 min 11-vs.-11 official football match.

	Team	Mean	SD	Mean diff (Δ%)	95% IC		ɳp^2^	*p*-value
					Lower	Upper		
Total distance (m)	SP	10,875	1,632.1	1,255 (11.5%)	−544.2	3,054.9	0.138	0.160
	RC	9,620	1,707.7					
Distance at 18–21 km·h^−1^ (m)	SP	404	142.0	53 (13.1%)	−100.5	206.2	0.038	0.470
	RC	351	141.6					
Distance at 21–24 km·h^−1^ (m)	SP	230	90.4	42 (18.3%)	−61.5	145.5	0.051	0.400
	RC	188	102.5					
Distance at 24–50 km·h^−1^ (m)	SP	161	94.7	56 (34.8%)	−29.7	141.9	0.123	0.180
	RC	105	52.5					
Sprints at 18–21 km·h^−1^ (n)	SP	22	7.8	−4 (−22.2%)	−4.5	4.4	0.000	0.980
	RC	26	5.7					
Sprints at 21–24 km·h^−1^ (n)	SP	13	5.0	0 (0.0%)	−5.6	0.8	0.161	0.120
	RC	13	6.1					
Sprints at 24–50 km·h^−1^ (n)	SP	8	4.5	0 (0%)	−2.1	0.2	0.193	0.090
	RC	8	3.4					
Sprints at 75–85%max (n)	SP	10	3.9	0 (0.0%)	−12.4	2.7	0.121	0.190
	RC	10	4.3					
Sprints at 85–95%max (n)	SP	4	2.4	−2 (−66.7%)	−5.8	6.2	0.000	0.950
	RC	6	3.6					
Sprints at 95–100% (n)	SP	1	0.7	−1 (−71.4%)	−4.1	4.7	0.002	0.880
	RC	2	1.4					
Accelerations (n)	SP	3,656	382.5	474 (13.0%)	−119.7	106.8.0	0.173	0.110
	RC	3,182	713.7					
Decelerations (n)	SP	3,681	392.3	502 (13.6%)	−82.7	1,086.3	0.195	0.090
	RC	3,179	690.8					
Aceleration load (AU)	SP	1,482	180.5	222 (15.0%)	5.7	438.5	0.257	**0.050** *
	RC	1,260	223.9					
Desaceleration load (AU)	SP	1,483	180.8	223 (15.0%)	5.8	440.0	0.257	**0.050** *
	RC	1,260	224,8					
Accelerations >3 m·s^−2^ (n)	SP	52	15.2	23 (44.6%)	10.5	36.2	0.520	**0.000** *
	RC	29	4.6					
Decelerations ≤3 m·s^−2^ (n)	SP	69	24.9	22 (31.6%)	−0.2	43.6	0.244	**0.050** *
	RC	47	11.4					
Accelerations >3 m·s^−2^ (m)	SP	384	136.3	174 (45.2%)	54.7	292.5	0.412	**0.010** *
	RC	210	59.0					
Decelerations ≤3 m·s^−2^ (m)	SP	483	176.4	208 (43.0%)	56.1	359.3	0.381	**0.011** *
	RC	275	66.4					

SP, semi-professional level; RC, recreational level; m, meters; n, number of efforts.

* Significant difference; acceleration load and deceleration load represent the mechanical load associated with acceleration and deceleration efforts, respectively. These values are calculated from WIMU accelerometer data in arbitrary units (AU), based on the magnitude and duration of acceleration events using proprietary algorithms by RealTrack Systems. Effect sizes (eta squared, *η*^2^): 0 < *η*^2^ < 0.04 “trivial”, 0.04 ≤ *η*^2^ ≤ 0.24 “small”, 0.25 ≤ *η*^2^ < 0.64 “moderate”, and *η*^2^ ≥ 0.64 “large.”

Tables 4–6, present the comparison between groups regarding the cardiorespiratory performance of during 90 min 11-vs.-11 official football match, particularly the response of the various components of HR to during 90 min 11-vs.-11 official football match. The physiological responses were divided into 1st half (1H) and 2nd half (2H). Baseline HR (heart rate up to 20 min before the match) and HR reserve were also measured, based on baseline HR and not based on resting HR, as suggested by some authors (22). The mean pre-match baseline HR was 17% lower in SP than RC group (*p* = 0.003; ɳp^2^: 0.475; moderate effect). Likewise, mean HR reserve was 12% higher in SP than RC players (*p* = 0.002; ɳp^2^: 0.–0.551; moderate effect). There were no other differences between groups in the 1st half (1H).

**Table 4:** Comparison between SP and RC groups of physiological performance values during 90 min 11-vs.-11 official football match.

	Team	Mean	SD	Mean diff (Δ%)	95% IC		ɳp^2^	*p*-value
					Lower	Upper		
Maximal HR (bpm)	SP	187	7.9	14 (7.5%)	−2.5	30.7	0.191	0.090
	RC	173	21.6					
Mean HR (bpm)	SP	149	14.9	8 (5.4%)	−12.6	28.9	0.049	0.412
	RC	141	23.7					
Mean HR (%max )	SP	80	6.1	−1 (−1.9%)	−7.8	4.9	0.017	0.630
	RC	81	5.5					
High HR (>85%) (m)	SP	6,487	1,960.2	2,657 (41.0%)	210.9	5,103.1	0.279	**0.035** *
	RC	3,830	2,612.9					
High HR (>85%) (n)	SP	28	9.9	3 (11.2%)	−12.5	18.6	0.013	0.679
	RC	25	18.8					
HR [70–80%] (m)	SP	1,570	619.7	−138 (−8.8%)	−1,016.7	7,405	0.008	0.741
	RC	1,708	1,014.7					
HR [80–90%] (m)	SP	3,201	940.2	−69 (−2.2%)	−1,327.4	1,188.2	0.001	0.907
	RC	3,270	1,407.6					
HR [90–95%] (m)	SP	2,613	1,059.9	907 (34.7%)	−228.8	2,042.1	0.173	0.109
	RC	1,706	1,037.8					
HR [95–200%] (m)	SP	1,697	860.6	590 (34.8%)	−264.2	1,444.0	0.136	0.161
	RC	1,107	685.1					
HR [70–80%] (n)	SP	37	15.5	6 (17.7%)	−14.1	27.4	0.033	0.502
	RC	31	23.1					
HR [80–90%] (n)	SP	66	16.0	11 (17.0%)	−14.9	37.5	0.058	0.371
	RC	55	32.1					
HR [90–95%] (n)	SP	88	27.0	23 (26.8%)	−15.6	63.1	0.107	0.217
	RC	65	46.0					
HR [95–200%] (n)	SP	46	22.0	17 (37.8%)	−6.4	41.2	0.150	0.139
	RC	29	22.1					

HR, heart rate; SP, semi-professional level; RC, recreational level; bpm, beats per minute; m, meters; n, number of efforts.

* Significant difference; effect sizes (eta squared, *η*^2^): 0 < *η*^2^ < 0.04 “trivial”, 0.04 ≤ *η*^2^ ≤ 0.24 “small”, 0.25 ≤ *η*^2^ < 0.64 “moderate”, and *η*^2^ ≥ 0.64 “large.”

**Table 5:** Comparison between SP and RC groups of physiological performance values in the 1st half (1H).

	Team	Mean	SD	Mean diff (Δ%)	95% IC		ɳp^2^	*p*-value
					Lower	Upper		
HR max (bpm)	SP	182	4.6	−2 (−1%)	−16.9	12.9	0.989	0.778
	RC	184	5.2					
Mean HR (bpm)	SP	151	6.8	−9 (−6%)	−31.6	12.6	0.968	0.372
	RC	160	7.7					
Mean HR (%max)	SP	81	5.3	−12 (13%)	−29.7	4.7	0.057	0.142
	RC	93	6.0					
High HR (>85% max) (m)	SP	3,384	499.7	177 (5%)	−1,443.6	1,797.3	0.944	0.818
	RC	3,207	566.6					
High HR (>85% max) (n)	SP	13	2.5	−3 (−19%)	−11.7	4.6	0.696	0.361
	RC	16	2.9					
HR [60–70]% (m)	SP	370	77.9	99 (27%)	−154.3	350.9	0.004	0.418
	RC	271	88.3					
HR [70–80]% (m)	SP	820	158.5	236 (29%)	−277.3	750.7	0.696	0.340
	RC	584	179.7					
HR [80–90]% (m)	SP	1,620	296.9	−487 (−23%)	−1,449.1	476.1	0.403	0.297
	RC	2,107	336.6					
HR [90–95]% (m)	SP	1,375	250.8	382 (28%)	−431.9	1,194.4	0.047	0.332
	RC	993	284.4					
HR [95–200]% (m)	SP	893	278.7	45 (5%)	−858.7	948.7	0.430	0.916
	RC	848	316.0					
HR [70–80]% (n)	SP	18	3.7	5 (28%)	−6.7	17.3	0.737	0.360
	RC	13	4.2					
HR [80–90]% (n)	SP	33	4.8	−2 (−6%)	−17.3	13.6	0.077	0.802
	RC	35	5.4					
HR [90–95]% (n)	SP	44	8.8	−2 (−4%)	−30.6	26.5	0.466	0.878
	RC	46	10.0					
HR [95–200]% (n)	SP	22	5.4	1 (5%)	−15.9	19.2	0.067	0.842
	RC	21	6.1					
HR_baseline_ (bpm)	SP	59	2.3	−12 (−17%)	−19.5	−4.8	0.475	**0.003** *
	RC	71	2.6					
HR_reserve_ (bpm)	SP	132	2.5	−16 (12%)	7.5	23.6	0.551	**0.002** *
	RC	116	2.8					

HR, heart rate; 1H, first half [0ʹ–45ʹ]; SP, semi-professional level; RC, recreational level; bpm, beats per minute; m, meters; n, number of efforts.

* Significant difference; effect sizes (eta squared, *η*^2^): 0 < *η*^2^ < 0.04 “trivial”, 0.04 ≤ *η*^2^ ≤ 0.24 “small”, 0.25 ≤ *η*^2^ < 0.64 “moderate”, and *η*^2^ ≥ 0.64 “large.”

**Table 6:** Comparison between SP and RC groups of physiological performance values in the 2nd half (2H).

	Team	Mean	SD	Mean diff (Δ%)	95% IC		ɳp^2^	*p*-value
					Lower	Upper		
HR max (bpm)	SP	185	3.1	1 (1%)	−9.3	10.6	0.995	0.890
	RC	184	3.5					
Mean HR (bpm)	SP	152	5.8	−2 (−1%)	−20.7	16.7	0.975	0.819
	RC	154	6.5					
Mean HR (%max)	SP	81	5.6	−15 (−16%)	−33.3	3.0	0.004	0.096
	RC	96	6.4					
High HR (>85% max) (m)	SP	3,103	421.4	858 (28%)	−507.9	2,224.8	0.942	0.199
	RC	2,245	477.8					
High HR (>85% max) (n)	SP	15	3.3	1 (1%)	−9.3	12.4	0.612	0.768
	RC	14	3.8					
HR [60–70]% (m)	SP	290	89.1	−96 (25%)	−384.7	193.4	0.115	0.490
	RC	386	101.1					
HR [70–80]% (m)	SP	749	144.5	−119 (−14%)	−588.2	349.2	0.482	0.593
	RC	868	163.9					
HR [80–90]% (m)	SP	1,580	172.1	480 (31%)	−78.1	1,038.2	0.510	0.086
	RC	1,100	195.2					
HR [90–95]% (m)	SP	1,238	171.5	698 (56%)	141.4	1,253.4	0.035	0.018
	RC	541	194.4					
HR [95–200]% (m)	SP	803	182.6	362 (45%)	−229.5	954.5	0.667	0.210
	RC	441	207.0					
HR [70–80]% (n)	SP	19	4.2	−13 (−41%)	−27.1	0.1	0.694	0.052
	RC	32	4.8					
HR [80–90]% (n)	SP	33	4.1	−1 (−1%)	−14.0	12.3	0.196	0.888
	RC	34	4.6					
HR [90–95]% (n)	SP	45	6.2	18 (40%)	−2.1	37.9	0.356	0.075
	RC	27	7.0					
HR [95–200]% (n)	SP	24	4.0	13 (54%)	−0.3	25.5	0.341	**0.050** *
	RC	11	4.5					

HR, heart rate; 2H, second half [45ʹ–90ʹ]; SP, semi-professional level; RC, recreational level; bpm, beats per minute; m, meters; n, number of efforts.

* Significant difference; effect sizes (eta squared, *η*^2^): 0 < *η*^2^ < 0.04 “trivial”, 0.04 ≤ *η*^2^ ≤ 0.24 “small”, 0.25 ≤ *η*^2^ < 0.64 “moderate”, and *η*^2^ ≥ 0.64 “large.”

In the 2nd half (2H) (Table 5), the differences between groups in the distance covered (m) at submaximal HR (90%–95% HR) stand out, with the SP group presenting higher distance (∼56%) and the number of efforts (∼54%). Regarding the number of races performed at the 70%–80% HR, the RC group presented a higher value (∼41%).

Tables 7, 8 show the correlation values between physical activity volume (min) and physiological performance indicators, in the 1st half (1H) and 2nd half (2H), respectively. The main associations were verified in the moderate to vigorous physical activity, with moderate correlations with the mean HR (bpm); distance traveled (m) with high HR and in the indicator, distance traveled (m) in the HR range 60%–70%, with moderate correlations between this and the levels of very vigorous physical activity; moderate to vigorous and the average of moderate to vigorous physical activity per day (min·day^−1^).

**Table 7:** Associations between physical activity volume (min) and HR components at 1st half.

		%Light PA (training)	%Moderate PA (training)	%Vigorous PA (training)	%Very vigorous PA (training)	%MVPA (training)
HR max (bpm) ^a^^NP^	Coef.	−0.166	−0.417	−0.267	−0.143	−0.412
	Sig.	0.540	0.108	0.318	0.598	0.113
Mean HR (bpm) ^a^^NP^	Coef.	−0.259	−0.402	−0.144	−0.113	−0.344
	Sig.	0.333	0.123	0.595	0.676	0.192
Mean HR (%max) ^a^^NP^	Coef.	−0.451	**−0.705**	−0.294	0.068	**−0.623**
	Sig.	0.080	**0.002** *	0.270	0.802	**0.010** *
High HR (>85% max) (m) ^a^^NP^	Coef.	0.044	−0.365	−0.093	−0.227	−0.295
	Sig.	0.870	0.164	0.732	0.398	0.268
High HR (>85% max) (n) ^a^^NP^	Coef.	−0.146	0.170	0.220	−0.003	0.220
	Sig.	0.589	0.530	0.414	0.990	0.412
HR [60–70] % (m)	Coef.	0.160	0.184	0.347	**0.770**	0.290
	Sig.	0.554	0.496	0.187	**0.000** *	0.276
HR [70–80] % (m)	Coef.	0.306	0.286	−0.123	0.119	0.138
	Sig.	0.249	0.282	0.649	0.660	0.609
HR [80–90] % (m)	Coef.	−0.177	−0.020	0.149	0.238	0.057
	Sig.	0.512	0.942	0.581	0.376	0.834
HR [90–95] % (m)	Coef.	0.225	−0.235	−0.066	−0.202	−0.193
	Sig.	0.403	0.381	0.808	0.453	0.475
HR [95–200] % (m)	Coef.	0.015	−0.295	−0.132	−0.238	−0.265
	Sig.	0.955	0.268	0.627	0.376	0.322
HR [70–80] % (n)	Coef.	0.333	0.199	−0.444	−0.352	−0.073
	Sig.	0.207	0.461	0.085	0.181	0.787
HR [80–90] % (n) ^a^^NP^	Coef.	0.135	−0.028	−0.120	−0.363	−0.076
	Sig.	0.617	0.917	0.657	0.167	0.779
HR [90–95] % (n)	Coef.	−0.031	−0.228	0.013	−0.232	−0.151
	Sig.	0.909	0.395	0.963	0.387	0.577
HR [95–200] % (n)	Coef.	−0.048	−0.221	−0.077	−0.219	−0.188
	Sig.	0.861	0.411	0.777	0.415	0.486
HR_baseline_ (bpm)	Coef.	0.307	−0.238	−0.272	−0.215	−0.210
	Sig.	0.248	0.375	0.308	0.424	0.435
HR_reserve_ (bpm)	Coef.	0.349	0.126	−0.311	−0.305	−0.061
	Sig.	0.186	0.643	0.240	0.251	0.823

HR, heart rate.

^a^ NP, non-parametric; bpm, beats per minute; m, meters; n, number of efforts.

* Significant correlation.

**Table 8:** Associations between physical activity volume (min) and HR components at 2nd half.

		%Light PA (training)	%Moderate PA (training)	%Vigorous PA (training)	%Very vigorous PA (training)	%MVPA (training)
HRmax (bpm)	Coef.	−0.046	−0.205	−0.113	−0.133	−0.195
	Sig.	0.867	0.445	0.676	0.623	0.470
Mean HR (bpm)	Coef.	−0.184	−0.254	−0.328	−0.244	−0.330
	Sig.	0.494	0.342	0.214	0.363	0.212
Mean HR (%max)	Coef.	**−0.543**	**−0.665**	−0.394	−0.049	**−0.643**
	Sig.	**0.030** *	**0.005** *	0.131	0.858	**0.007** *
High HR (>85% max) (m)	Coef.	0.205	−0.293	−0.371	−0.233	−0.376
	Sig.	0.446	0.271	0.158	0.386	0.151
High HR (>85% max) (n)	Coef.	0.326	**0.520**	0.171	−0.080	0.438
	Sig.	0.218	**0.039** *	0.527	0.770	0.090
HR60–70] % (m)	Coef.	−0.186	−0.144	0.206	0.173	−0.002
	Sig.	0.492	0.596	0.445	0.521	0.995
HR [70–80] % (m)	Coef.	−0.089	0.122	0.348	**0.533**	0.248
	Sig.	0.744	0.653	0.186	**0.033** *	0.354
HR [80–90] % (m)	Coef.	0.446	0.281	−0.006	−0.028	0.190
	Sig.	0.083	0.292	0.984	0.918	0.480
HR [90–95] % (m)	Coef.	**0.499**	0.032	−0.174	−0.250	−0.060
	Sig.	**0.049** *	0.906	0.519	0.351	0.824
HR [95–200] % (m)	Coef.	0.283	0.033	−0.212	−0.265	−0.077
	Sig.	0.287	0.902	0.430	0.321	0.776
HR [70–80] % (n)	Coef.	−0.408	−0.139	0.283	0.341	0.038
	Sig.	0.116	0.607	0.289	0.196	0.889
HR [80–90] % (n)	Coef.	0.005	0.103	0.003	−0.142	0.073
	Sig.	0.986	0.703	0.991	0.600	0.789
HR [90–95] % (n)	Coef.	0.292	0.035	−0.230	−0.324	−0.084
	Sig.	0.272	0.898	0.392	0.221	0.756
HR [95–200] % (n)	Coef.	0.222	−0.058	−0.268	−0.278	−0.167
	Sig.	0.408	0.831	0.315	0.297	0.537

HR, heart rate; bpm, beats per minute; m, meters; n, number of efforts.

* Significant correlation.

It is important to highlight, in the associations of the 1st half (1H), the strong correlation between the %moderate PA in training and the mean HR (% max.) and between the %very vigorous PA in training and the distance traveled (m) in the interval 60%–70% max HR. Between moderate to vigorous %PA and mean HR (% max.), a moderate correlation was found.

In the 2nd half (2H), the main associations were between mean HR (%max.) and %mild PA; %Moderate PA and % moderate to vigorous PA during the training period. All correlations found are of moderate strength. There were also moderate correlations between the number of efforts in high HR conditions and %moderate PA in training; distance traveled (m) in the range 60%–70% HRmax. and % very vigorous PA in training; distance traveled (m) in the range 90%–95% HRmax. and % light PA in training.

## Discussion

Regarding health and physiological indicators related to match performance, our results suggest that recreational practitioners (∼2 workouts·week^−1^ and 1 match) are subject to a very high internal load for much lower external load values when compared to Semi-Professional players (∼4 workouts·week^−1^ and 1 match). The differences between groups were observed in the total distance in the match with high HR (>85%) and HR between 90% and 95%. The semiprofessional team also attained a higher number of maximal HR efforts (95%–100%) (15, 24, 25). Relating to training physical activity (in percentage), the SP assumed higher values (∼33%) of very vigorous physical activity when compared to the RC group.

The HR represents a non-invasive method that is frequently used for monitoring the physiological response in team sports (15). As expected, the SP group, with greater training volume, frequency, and probably even intensity, showed superior cardiorespiratory adaptations, reflected by a 17% lower resting heart rate and 12% higher HR reserve. A lower resting HR is common in well-conditioned athletes and reflects a more efficient heart that pumps more blood with fewer beats. This adaptation suggest increased stroke volume, enhanced oxygen delivery, and improved vascular function 6, 7, 26, 27). As a result, heart rate reserve increases, indicating better cardiorespiratory fitness, greater ability to sustain high intensities for longer durations (as the heart is more efficient), and faster recovery between sprints and plays. A higher HR reserve translates into a wider safe working zone for training and competition—that is, it allows for higher intensities with the same or even lower internal load, which in turn represents a competitive advantage. In this regard, applying additional stimuli to already-fatigued recreational players with limited training frequency may not yield the intended benefits (15, 18, 21, 27).

Consistent with previous research, HR values during the match remained high for both groups, averaging ∼80%–81% of HRmax, with ∼65% of playing time spent between 70% and 90% HRmax (15). This confirms the intermittent nature of football, alternating short, intense anaerobic bursts (sometimes exceeding 100% HRmax) with brief aerobic recovery periods (24). The type of floor is also an environmental constraint that may have impacted the results found for the physical and physiological variables. From the perspective of exercise for health, understanding the varying demands of physical and physiological exercise for health promotion among different levels of football players is essential for customizing training regimens. Our results suggest some practical implication; for SP: training should focus on replicating match demands through high-intensity drills, interval training, and sport-specific strength and conditioning programs (28, 29). Also, recovery protocols should address the greater physiological strain experienced during matches (30). For RC players, training can prioritize general fitness improvements, basic tactical awareness, and injury prevention, with a less intense approach to accommodate diverse fitness levels and motivations (31–33).

Deceleration, directional changes, and reacceleration during football impose significant physiological demands, requiring rapid bodily adjustments, increasing oxygen consumption, and engaging both aerobic and anaerobic energy systems (8, 9, 15). These actions intensify muscle recruitment and elevate metabolic cost, which is reflected in heightened heart rate responses due to increased cardiorespiratorydemand (8, 9, 15). In fact, differences were observed between SP and RC for some high-intensity fitness variables, particularly in acceleration and deceleration load, distance covered during high accelerations (>3 m.s^−2^) and decelerations (≤3 m.s^−2^), and the number of these high accelerations and decelerations.

Our study had some limitations. First, we are aware that the sample size (*N* = 16) was small, which limits generalizability. However, while a larger sample is always desirable, the practical restrictions of working with a selective group make larger recruitment problematic. Also, typically, highly trained athletes have demanding calendars, making access for research challenging. Factors such as competition calendars, training commitments, and limited availability limited our capacity to assess more athletes within this population. Despite that, we made efforts to ensure our sample as representative as possible. Second, the study was cross-sectional, while longitudinal approaches would better capture seasonal fluctuations in load and performance. Third, complementary health indicators (e.g., blood pressure, lipid profile, glucose metabolism) were not measured, limiting conclusions about the cardiorespiratory impact for SP and RC groups. Fourth, physical and physiological demands were evaluated in a typical week of the competitive period and a unique match, though multiple observations across a season would increase reliability. Finally, environmental and contextual variables (e.g., pitch typer), as well as individual characteristics (e.g., playing position, age) (15), were not controlled during analysis, and in this regard physical and physiological demands of training and Official Football Match may differ. Future studies should consider these factors, whenever possible, and include a broader range of competitive levels, from recreational to elite.

## Conclusion

The physical and physiological requirements of training and official football matches vary considerably between recreational and semi-professional players, influenced by differences in match intensity, fitness level, and training structures. Semi-professional football players typically participate in a greater weekly volume of physical activity compared to recreation players, but lacking an alternative professional vocation. The augmented frequency of weekly training sessions (Semi-Professional) correlates with a heightened weekly volume of intense physical exercise. Semi-Professional football players engage in a greater volume of intense physical exercise during training and daily social interactions. A significant distinction between the Semi-Professional and recreational levels is the capacity to cover a greater distance during a 90-min competition while maintaining increased heart rate (HR) levels (>85%HRmax), particularly in the latter half (45–90 min). Despite a comparable number of attempts exceeding 85% HRmax, Semi-Professional practitioners covered a greater distance (m), indicating enhanced cardiorespiratory capability among those who engage in more frequent and intense exercise. This may also indicate the match's higher level of quality, resulting in more stress. The frequency of football training sessions correlates with improved cardiorespiratory capacity. This study's findings indicate that recreational athletes (about 2 sessions weekly and 1 match) experience significantly more internal stress relative to their external load compared to semi-professional players (around 4 exercises weekly and 1 match). In this sense, recreational players may require modified training protocols to optimize performance while managing internal load.

## Data Availability

The raw data supporting the conclusions of this article will be made available by the authors, without undue reservation.
